# Comparison between supraglottic airway devices and endotracheal tubes in patients undergoing laparoscopic surgery: A systematic review and meta-analysis

**DOI:** 10.1097/MD.0000000000004598

**Published:** 2016-08-19

**Authors:** Sun Kyung Park, Geum Ko, Geun Joo Choi, Eun Jin Ahn, Hyun Kang

**Affiliations:** a Department of Anesthesiology and Pain Medicine, College of Medicine; b Medical Course, Jeju National University School of Medicine, Jeju National University, Jeju; c Department of Anesthesiology and Pain Medicine, Chung-Ang University College of Medicine; d Department of Anesthesiology and Pain Medicine, Inje University Seoul Paik Hospital, Seoul, Korea.

**Keywords:** intubation, laparoscopy, laryngeal masks, supraglottic airway

## Abstract

**Background:**

Comparisons between the efficacies of supraglottic airway devices (SGAs) and endotracheal tubes (ETTs) in patients undergoing laparoscopic surgeries have yielded conflicting results. Therefore, in this meta-analysis, we compared the clinical performance and incidence of complications between SGAs and ETT intubation in laparoscopic surgery.

**Methods:**

A comprehensive search was conducted using MEDLINE, EMBASE, the Cochrane Central Register of Controlled Trials, and Google Scholar to identify randomized controlled trials that compared SGAs with ETTs in laparoscopic surgery.

**Results:**

In total, 1433 patients from 17 studies were included in the final analysis. SGAs and ETTs showed no difference in insertion success rate on the first attempt (relative risk [RR] 1.01, 95% confidence interval [CI] 0.99–1.03), insertion time (standardized mean difference 1.57, 95% CI −3.74 to 0.61), and oropharyngeal leak pressure (OLP) (mean difference −2.54, 95% CI −7.59 to 2.50). The incidence of desaturation (RR 3.65, 95% CI 1.39–9.62), gastric insufflations (RR 0.90, 95% CI 0.48–1.71), regurgitation (RR 0.98, 95% CI 0.02–49.13), and aspiration (RR 0.99, 95% CI 0.01–78.4) also showed no intergroup differences. However, the incidence of laryngospasm (RR 3.12, 95% CI 1.29–7.52), cough at removal (RR 6.68, 95% CI 4.70–9.48), dysphagia (RR 1.47, 95% CI 1.12–1.95) or dysphonia (RR 4.41, 95% CI 1.25–15.55), sore throat (RR 1.60, 95% CI 1.33–1.93), and hoarseness (RR 1.53, 95% CI 1.29–1.81) was higher in the ETT group than in the SGA group.

**Conclusions:**

The incidence of laryngospasm, cough at removal, dysphagia or dysphonia, sore throat, and hoarseness were higher in the ETT group than in the SGA group. However, the groups showed no differences in the rate of insertion success on the first attempt, insertion time, OLP, and other complications. Therefore, SGAs might be clinically more useful as effective airways in laparoscopic surgery.

## Abstract 2

Supplemental Digital Content is available in the text

## 1 Introduction

Supraglottic airway devices (SGAs) are widely used for routine general anesthesia and to secure the airway in cases of cardiac arrest or out-of-hospital emergencies.^[1]^ Moreover, the newly designed second-generation SGAs reduce the risk of aspiration, by providing a higher oropharyngeal leak pressure (OLP) than do first-generation SGAs. Although SGAs have advanced designs, their safety in laparoscopic surgery has been controversial.

Laparoscopy is popular and is being widely used in various fields of surgery. Its advantages are minimal invasiveness, reduced postoperative pain, and shorter hospital stay.^[2]^ However, intraoperative CO_2_ insufflation into the peritoneum increases the load on the respiratory system, and also the risk of air leakage, insufficient ventilation, or gastric insufflations, which results in aspiration or respiratory complications.^[3]^ Pneumoperitoneum during laparoscopic surgery shifts the diaphragm upwards, which increases the airway pressure. This increased airway pressure above the OLP leads to air leakage, insufficient ventilation, or gastric insufflations, thereby increasing the risk of regurgitation. The increased risk of regurgitation, in turn, increases the risk of aspiration.^[4,5]^

A few systematic reviews have compared the clinical performance of and complications involved in the use of SGAs and endotracheal tubes (ETTs) under general anesthesia. However, these studies were limited by their small sample sizes and the use of various types of surgery that were controversial.^[6,7]^ Further, these studies did not compare the clinical performance and complications of SGAs and ETTs in laparoscopic surgery, which is expected to have a different effect on pulmonary physiology and mechanics.

To our knowledge, no previous systematic reviews or meta-analyses have compared various SGAs and ETTs used in laparoscopic surgery. Therefore, we performed this systematic review and meta-analysis to compare the clinical performance of and complications associated with the use of various SGAs and ETTs in patients undergoing laparoscopic surgery by evaluating previous well-designed, controlled trials.

## 2 Materials and methods

The present systematic review and meta-analysis was registered in PROSPERO (CRD42015027996) and was conducted according to the guidelines of the Preferred Reporting Items for Systematic Reviews and Meta-Analysis (PRISMA).^[8]^

### 2.1 Literature search

Two authors (SKP and GJC) independently carried out database searches using MEDLINE, EMBASE, the Cochrane Central Register of Controlled Trials (CENTRAL), and Google Scholar in November 2015, and the data were updated in March 2016. Our study did not impose any language limitations. The reference lists of the identified and eligible articles were also searched manually for identifying more articles. The search strategy, which included a combination of free text, Medical Subject Headings, and EMTREE terms, is described in the Appendix.

### 2.2 Study selection

The inclusion and exclusion criteria were determined before the systematic search. Randomized controlled trials (RCTs) that compared the performance of and risk of complications associated with the use of SGAs and ETTs in patients receiving laparoscopic surgery under general anesthesia were included. Review articles, case reports, case-series, letters to the editor, commentaries, proceedings, laboratory science studies, and any other nonrelevant studies were excluded.

Two authors (SKP and GJC) independently scanned the titles and abstracts of the reports identified via the abovementioned search strategies. If a report was determined eligible from the title or abstract, the full paper was retrieved. Potentially relevant studies chosen by at least 1 author were retrieved, and full-text versions were evaluated. The same authors independently selected eligible studies and arrived at a consensus through discussion as to whether a study should be included or excluded. Any disagreement over the inclusion or exclusion of a study was settled through discussion with a third investigator (HK).

### 2.3 Data extraction

All inter-related data from the included studies were independently extracted and entered into standardized forms by 2 authors (SKP and GJC), and the data were then cross-checked. Any discrepancy was resolved through discussion. If an agreement could not be reached, the dispute was resolved through discussion with a third investigator (HK). The standardized form used in this study included the following items: title, name of first author, name of journal, year of publication, study design, registration of clinical trial, competing interest, country, risk of bias, inclusion criteria, exclusion criteria, sex, age, weight, height of patients, American Society of Anesthesiologists physical status, type of surgery, type of airway device, size of airway device, insertion time, insertion success rate at the first attempt, OLP, complications during anesthesia, and complications after anesthesia.

The data were initially extracted from tables or text. In case of studies with missing or incomplete data, an attempt was made to contact the study authors to obtain the relevant information. If the authors did not respond or did not have current information, the data were extracted from available figures.

### 2.4 Risk of bias assessment

The quality of studies was independently assessed by 2 authors (GK and EJA) by using the tool “risk of bias” in Review Manager (version 5.3; The Cochrane Collaboration, Oxford, UK). Quality was evaluated using the following potential sources of bias: sequence generation, allocation concealment, blinding of participants, outcome assessor during anesthesia and after anesthesia, incomplete data, and selective reporting. The methodology used in each study was graded as “high risk,” “low risk,” or “unclear,” depending on a high risk of bias, low risk of bias, or uncertain bias, respectively.

### 2.5 Statistical analysis

We conducted this meta-analysis by using Review Manager (version 5.3; The Cochrane Collaboration) and Comprehensive Meta-Analysis (version 2.0; Biostat, Englewood, NJ). Two authors (SKP and GJC) independently input all data to the software. The pooled relative risk (RR) or mean difference (MD) and their 95% confidence intervals (CIs) were calculated for each outcome. We used the chi-square test for assessing homogeneity and the I^2^ test for assessing heterogeneity. A level of 10% significance (*P* < 0.1) for the chi-square statistic or an I^2^ greater than 50% was considered to indicate considerable heterogeneity. A fixed-effects model was selected if the *P* value for the chi-square test was >0.1 and the I^2^ value was <50%. In cases in which the I^2^ value was >50%, random-effects model was used.^[9,10]^

Because the total number of studies that showed substantial heterogeneity was less than 10, t-statistics (Hartung-Knapp-Sidik-Jonkman method) were used instead of the Z-test in all random-effects analysis to lower the error rate.^[11]^ We calculated the number needed to treat (NNT) by using a 95% CI based on the absolute risk reduction as an estimate of the overall clinical impact of the intervention.^[12]^ Publication bias was assessed using Begg funnel plot and Egger linear regression test; a *P* value <0.05 was used to identify the presence of a publication bias, and the funnel plots for each data set were visually assessed for asymmetry.

### 2.6 Ethics statement

This study analyzed publicly available data, and thus protocol review and informed consent were unnecessary.

## 3 Results

### 3.1 Literature search and study characteristics

**Figure 1:**
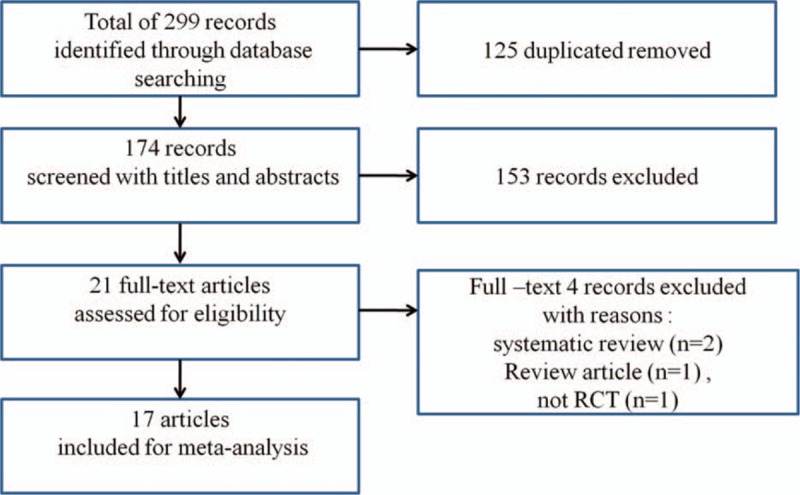
Flow chart of literature search and selection.

We searched 299 potentially relevant studies in the databases (Embase, MEDLINE, CENTRAL, and Google Scholar). After excluding 125 duplicates, 174 studies were screened using titles and abstracts; of these, 153 studies were excluded. After full-text assessment for eligibility, 4 studies were excluded because they were systematic reviews,^[13,14]^ a review article,^[15]^ or not a RCT.^[16]^ Finally, 17 studies were included in this systematic review and meta-analysis (Fig. 1). The study characteristics are summarized in Table 1.

**Table 1:**
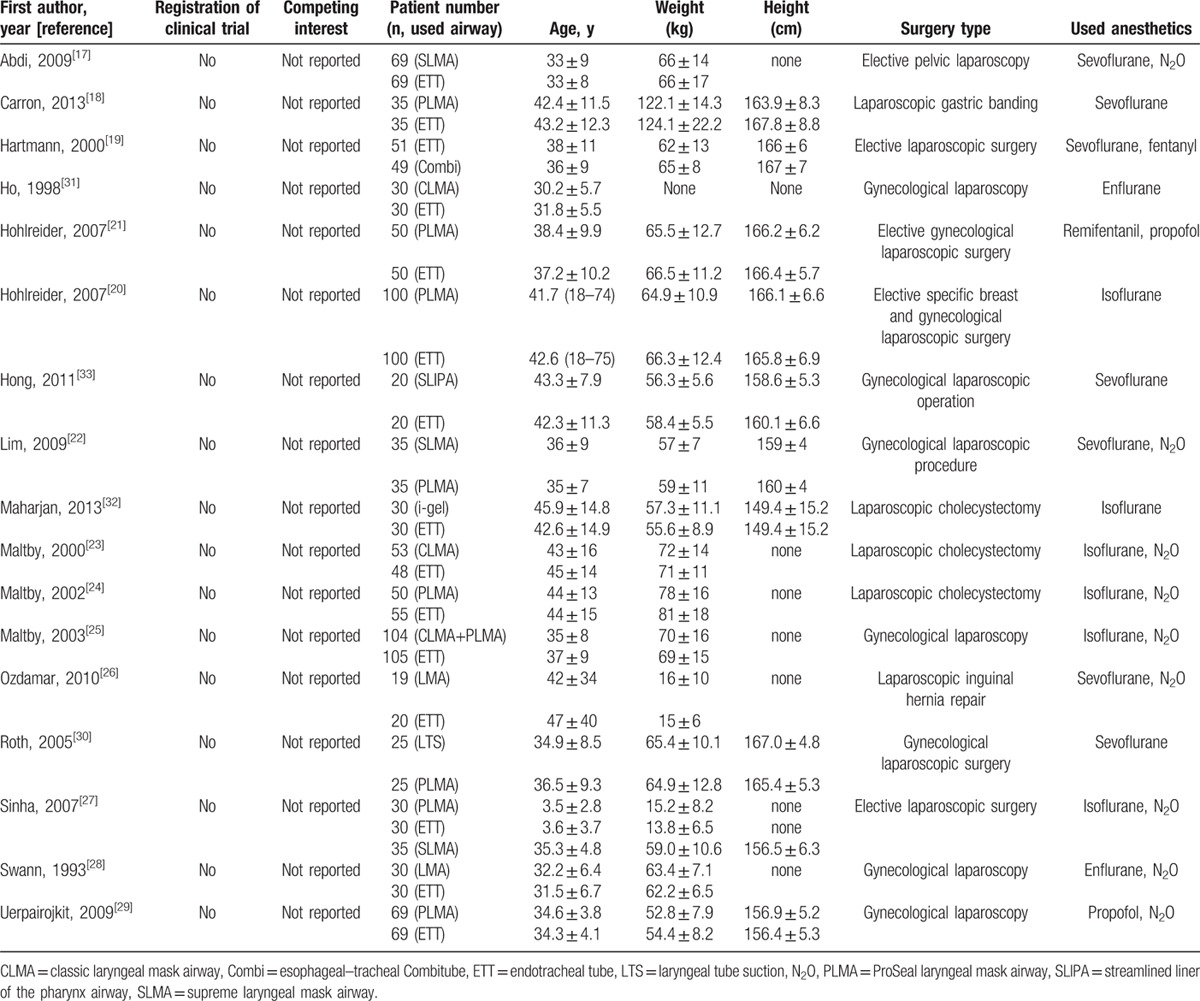
Study characteristics.

### 3.2 Risk of bias

Although all the selected studies mentioned randomization, only 13 studies described the method used for random sequence generation.^[17–29]^ Eight studies described allocation concealment.^[18–22,24,29,30]^ While blinding of participants was applied in 2 studies,^[18,20]^ 5 studies reported blinding the outcome assessors during operation^[17,20,22,26,28]^ and 6 studies reported blinding the outcome assessors after operation.^[17,18,20–22,31]^ None of the studies had incomplete outcome data. The overall risks of bias are shown in Table 2.

**Table 2:**
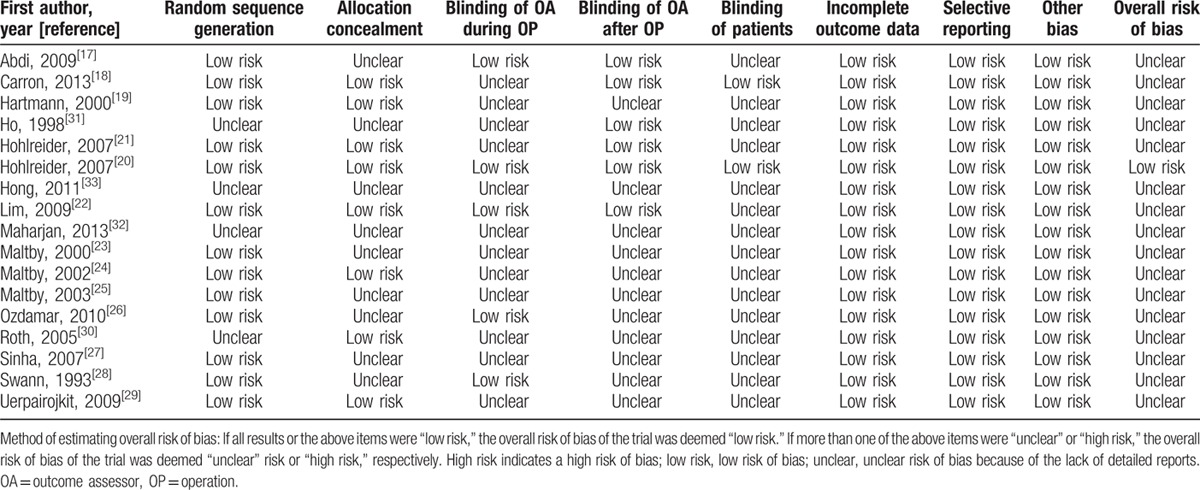
Risk of bias in the included randomized controlled trials.

### 3.3 Insertion success rate on the first attempt

The insertion success rate on the first attempt was compared in 6 studies (4 studies compared ProSeal laryngeal mask airway [PLMA] with ETT,^[20–22,27]^ 1 study compared Profile Soft-Seal with ETT,^[29]^ and 1 study compared esophageal-tracheal Combitube with ETT).^[19]^ The combined result showed no evidence of any difference between groups (RR 1.01, 95% CI 0.99–1.03, *P*_chi_^2^ = 0.23, I^2^ = 27%, NNT = 200). Subgroup analysis for PLMA showed no difference between the groups (RR 1.01, 95% CI 0.98–103, *P*_chi_^2^ = 0.16, I^2^ = 42%, NNT = 135).

### 3.4 Device insertion time

**Figure 2:**
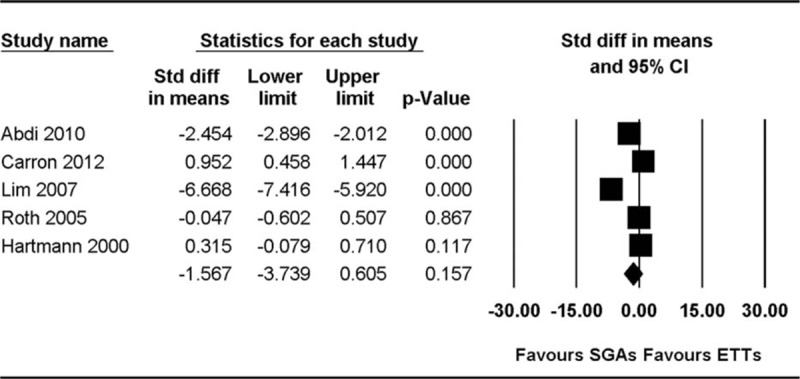
Forest plot showing the device insertion time.

Device insertion time was measured in 5 studies.^[17–19,22,30]^ The combined result showed substantial heterogeneity (standardized MD [SMD] 1.57, 95% CI −3.74 to 0.61, *P*_chi_^2^ < 0.001, *I*^2^ = 99%). However, the result remained unchanged even in the subgroup analysis performed for PLMA versus ETT (SMD 1.91, 95% CI −2.16 to 5.97, *P*_chi_^2^ < 0.001, *I*^2^ = 99%) (Fig. 2).

### 3.5 Oropharyngeal leak pressure

Two studies measured OLP by using the manometric stability technique.^[24,30]^ Overall, OLP showed no evidence of any difference between the groups (MD −2.54, 95% CI −7.59 to 2.50, *P*_chi_^2^ < 0.001, *I*^2^ = 99%).

### 3.6 Safety analyses

#### 3.6.1 Desaturation

The incidence of desaturation was compared in 5 studies.^[18,21,22,31,32]^ The combined results showed no evidence of any difference between the groups (RR 3.65, 95% CI 1.39–9.62, *P*_chi_^2^ = 0.996, *I*^2^ = 0.0%, NNT = 24).

#### 3.6.2 Laryngospasm and bronchoconstriction

**Figure 3:**
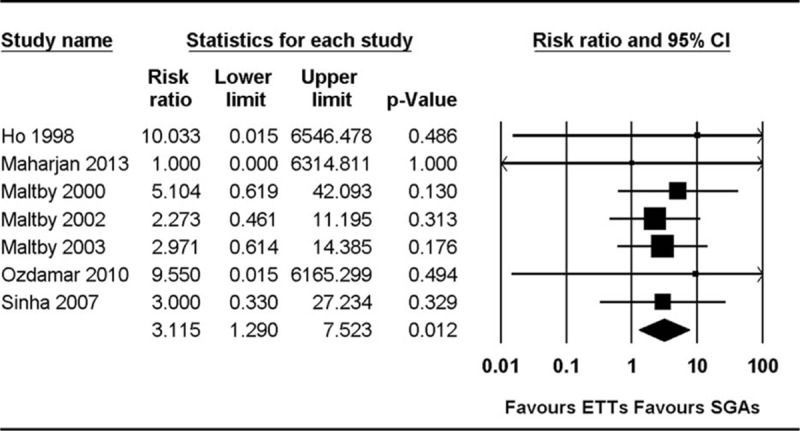
Forest plot showing laryngospasm.

The incidence of laryngospasm was compared in 7 studies.^[23–27,31,32]^ Pooled analysis showed that the incidence of laryngospasm was higher in the ETT group than in the SGA group (RR 3.12, 95% CI 1.29–7.52, *P*_chi_^2^ = 0.995, *I*^2^ = 0%, NNT = 21) (Fig. 3). Subgroup analysis based on the type of airway device used in 7 studies that compared ETT with PLMA or classic LMA (CLMA) showed that the incidence of laryngospasm was lower in the PLMA or CLMA group than in the ETT group (RR 3.36, 95% CI 1.34–8.47, *P*_chi_^2^ = 0.988, *I*^2^ = 0%, NNT = 19). Bronchoconstriction was reported in 1 study, which reported a single case involving the use of ETT and none involving the use of PLMA.^[18]^

#### 3.6.3 Blood staining

The incidence of blood staining on devices was compared in 8 studies.^[18–22,27,29,33]^ There was no evidence of any difference in the incidence of blood staining on devices (RR 0.85, 95% CI 0.41–1.79, *P*_chi_^2^ = 0.04, *I*^2^ = 50%, NNT = 2987). Subgroup analysis based on the type of airway device used in 6 studies that compared ETT with PLMA also showed no evidence of any difference (RR 0.87, 95% CI 0.34–2.22, *P*_chi_^2^ = 0.02, *I*^2^ = 61%, NNT = 399).

#### 3.6.4 Sore throat

**Figure 4:**
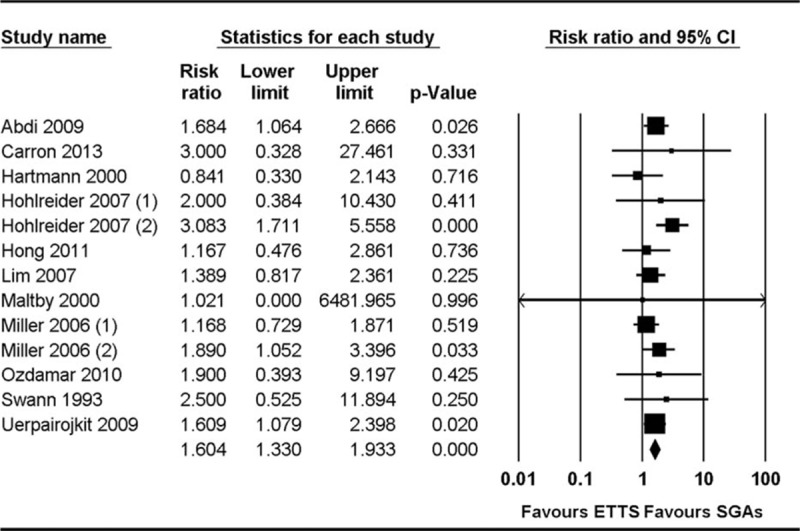
Forest plot showing sore throat.

The incidence of sore throat was compared in 13 studies.^[17–26,28,29,33]^ Pooled analysis showed that the incidence of sore throat was higher in the ETT group than in the SGA group (RR 1.60, 95% CI 1.33–1.93, *P*_chi_^2^ = 0.603, *I*^2^ = 0%, NNT = 10) (Fig. 4). Subgroup analysis based on the type of airway device used in 7 studies that compared ETT with PLMA showed that the incidence of sore throat was lower in the PLMA group than in the ETT group (RR 1.82, 95% CI 1.42–2.39, *P*_chi_^2^ = 0.458, *I*^2^ = 0%, NNT = 7).

#### 3.6.5 Hoarseness

The incidence of hoarseness was compared in 8 studies.^[17,19,21,26,28,29,31,33]^ Pooled analysis showed that the incidence of hoarseness was higher in the ETT group than in the SGA group (RR 1.53, 95% CI 1.29–1.81, *P*_chi_^2^ = 0.089, *I*^2^ = 43%, NNT = 7).

#### 3.6.6 Dysphagia and dysphonia

The incidence of dysphagia was compared in 6 studies,^[17,19–21,23,29]^ and the incidence of dysphonia was compared in 3 studies.^[18,20,21]^ Pooled analyses showed that the incidence of dysphagia and dysphonia were higher in the ETT group than in the SGA group (for dysphagia: RR 1.47, 95% CI 1.12–1.95, *P*_chi_^2^ = 0.37, *I*^2^ = 8%, NNT = 15; for dysphonia: RR 4.41, 95% CI 1.25–15.55, *P*_chi_^2^ = 0.916, *I*^2^ = 0%, NNT = 19).

#### 3.6.7 Gastric insufflation

**Figure 5:**
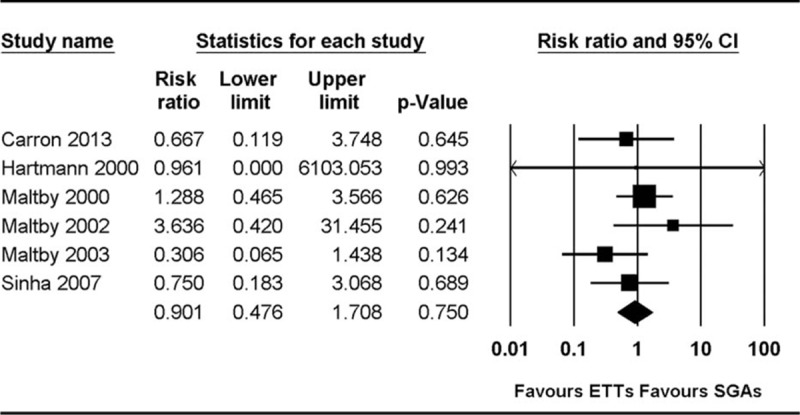
Forest plot showing gastric insufflation.

The incidence of gastric insufflation was compared in 6 studies.^[18,19,23–25,27]^ Three studies reported gastric insufflation by using the following scale: decrease of 1 to 2, increase of 0 to 2, or increase of 3 to 6 on a 0 to 10 scale (0 = empty stomach, and 10 = distension that interfered with surgical exposure).^[23–25]^ To standardize the scale, we considered an increase of 3 to 6 on the 0 to 10 scale to indicate gastric insufflation. The combined results showed no evidence of any difference between the groups (RR 0.90, 95% CI 0.48–1.71, *P*_chi_^2^ = 0.530, *I*^2^ = 1.3%, NNT = 119) (Fig. 5).

#### 3.6.8 Regurgitation and aspiration

The incidence of regurgitation was compared in 5 studies,^[19,26,27,32,33]^ and the incidence of aspiration was compared in 4 studies.^[18,26,27,32]^ There were no reports with respect to regurgitation or aspiration. The combined results showed no evidence of any differences between the groups with respect to regurgitation (RR 0.98, 95% CI 0.02–49.13, *P*_chi_^2^ = 1.00, *I*^2^ = 0%, NNT = NA) and aspiration (RR 0.99, 95% CI 0.01–78.4, *P*_chi_^2^ = 1.00, *I*^2^ = 0%, NNT = NA).

#### 3.6.9 Nausea and vomiting

The incidence of nausea was compared in 5 studies,^[20,21,27–29]^ and the incidence of vomiting was compared in 8 studies.^[18,20,21,23,24,26,28,29]^ The combined results showed no evidence of any differences between the groups with respect to nausea (RR 1.52, 95% CI 0.56–4.07, *P*_chi_^2^ = 0.000, *I*^2^ = 84.01%, NNT = 9) and vomiting (RR 1.93, 95% CI 0.97–3.87, *P*_chi_^2^ = 0.136, *I*^2^ = 36.73%, NNT = 20).

#### 3.6.10 Cough at removal and hiccup

The incidence of cough at removal was compared in 7 studies,^[18,23–27,31]^ and hiccup was compared in 1 study.^[31]^ Pooled analysis showed that the incidence of cough at removal was higher in the ETT group than in the SGA group (RR 6.68, 95% CI 4.70–9.48, *P*_chi_^2^ = 0.168, *I*^2^ = 34.0%, NNT = 2). Hiccup was reported only in 1 study, which documented 2 cases involving the use of ETT and no case involving the use of PLMA.

#### 3.6.11 Bradycardia

The incidence of bradycardia was compared in 2 studies.^[18,31]^ The combined results showed no evidence of any difference between the groups (RR 0.23, 95% CI 0.001–41.23, *P*_chi_^2^ = 0.678, *I*^2^ = 0.0%, NNT = 65).

### 3.7 Publication bias

No evidence of publication bias was detected by Egger linear regression test or Begg funnel plot. None of the *P* values obtained using Egger regression test was <0.1, which indicated a publication bias.

## 4 Discussion

In our meta-analysis, the incidence of laryngospasm, dysphagia or dysphonia, sore throat, cough at removal, and hoarseness were higher in the ETT group than in the SGA group. However, we could not find any difference between the ETT and SGA groups in the insertion success rate on the first attempt, insertion time, OLP, incidence of desaturation, gastric insufflation, regurgitation, and blood staining on the device.

Various types of SGAs have been introduced into clinical practice. The first-generation SGAs were “simple airway devices” and included the CLMA, Cobra perilaryngeal airway, laryngeal tube, and other standard LMAs. Because the Cobra perilaryngeal airway and the laryngeal tube without a drain tube (DT) do not have a part designed to protect against aspiration, they were first-generation SGAs, even though their design differs from that of the CLMA. The second-generation SGAs have a specific design (such as a DT) to minimize the risk of aspiration caused by the reflux of gastric contents, and they include the PLMA, supreme LMA (SLMA), laryngeal tube suction II (LTS-II) and its disposable version (LTS-D), esophageal blocker, i-gel, and streamlined liner of the pharynx airway (SLIPA).^[15,34]^ Many studies have proven the clinical efficacy and usefulness of SGAs in laparoscopic surgeries, and there has been a paradigm shift from ETTs to SGAs. However, as ETT intubation has been proven an effective method of securing the airway in laparoscopic surgery, surgeons hesitate to use SGAs in laparoscopic surgeries because of safety concerns.^[35]^ Further, although many studies have compared the clinical performances and complications of SGAs and ETTs, there is controversy surrounding which is the more appropriate airway for laparoscopic surgery.

Studies on SGAs have defined OLP as the degree of airway protection and the feasibility for positive pressure ventilation.^[36]^ Maintaining appropriate OLP is indispensable when using SGAs in laparoscopic surgery to protect the airway and maintain adequate ventilation. Small-scale comparative studies have shown similar OLPs when using SGAs and ETTs, and this finding supports the use of SGAs in laparoscopic surgery from the perspective of providing a reliable airway. This finding is also consistent with that of a recent study demonstrating that SGAs could ensure efficient OLP to seal the airway in laparoscopic surgery.^[15]^ However, since only second-generation SGAs containing a ventilation tube and gastric access via a DT were included in this meta-analysis, and since the number of included studies was less, the results are controversial. Nevertheless, this finding is consistent with that of a study demonstrating that various types of SGAs could ensure efficient OLP to seal the airway in a cadaveric model of elevated esophageal pressure. In particular, devices with additional DTs tend to decrease the risk of aspiration.^[37]^

Further, safety issues, including incidences of desaturation and regurgitation, were similar between the ETT and SGA groups. As desaturation can be an indicator of maintenance of adequate ventilation, this result supports the fact that appropriate ventilation was achieved with both devices without any leakage during CO_2_ insufflation. Moreover, no regurgitation and aspiration were reported in any of the studies. Further, as the lower esophageal sphincter pressure shows an adaptive increase during pneumoperitoneum, the risk of regurgitation does not increase in laparoscopic surgery.^[38]^ Thus, we can presume that SGAs are effective in preventing aspiration during elective laparoscopic surgery.

The insertion success rate on the first attempt and insertion time were similar between the groups, suggesting that SGAs were as effective as ETTs for general anesthesia in laparoscopic surgery. However, we could not resolve the controversy because this study included a small number of subjects, and because of differences in insertion methods and the experience of the anesthesiologists.

Sore throat, dysphagia, and dysphonia are complications associated with anatomical regions (namely, the pharynx and hypopharynx), where a high cuff pressure is applied.^[39,40]^ Additionally, the irritation of the vocal cords and laryngotracheal mucosa resulted in hoarseness, laryngospasm, and cough.^[7,41]^ In this study, the incidence of sore throat, hoarseness, dysphagia, dysphonia, laryngospasm, and cough were lower in the SGA group than in the ETT group. This finding indicates that, compared with ETTs, SGAs induced lesser trauma on the vocal cords and trachea, and lesser pressure damage to the pharynx. Because the cuffs of SGAs are placed superior to the larynx, they cause less irritation to the vocal cords and trachea.^[7]^ Additionally, Ulrich-Pur et al^[42]^ demonstrated that most SGAs (except intubating LMA) showed an appropriate pressure to maintain mucosal perfusion when using the cuff pressure recommended by the manufacturers.

We could not find any difference between ETT and Combitube in terms of dysphagia and sore throat.^[43]^ As Combitube applies higher cuff pressure on the oropharyngeal mucosa if applied for a long time,^[44,45]^ it may increase the incidence of dysphagia and sore throat. The incidence of these complications is also similar between Combitube and ETT. Nevertheless, this result should be interpreted cautiously because it is the finding of just 1 study; additional studies are needed to confirm this finding and ascertain its applicability.

The present study has several limitations. First, SGAs have different designs to protect against aspiration and to maintain ventilation. Further, the evaluated studies have a number of potential sources of clinical and methodological heterogeneity. Although we conducted subgroup and sensitivity analyses to try to control for some of these factors, we could not account for all possible confounding factors when designing the study.

Second, there is the possibility of publication bias derived from studies that are not published in the current literature because of null results or small sample sizes. However, on the basis of the results of Begg funnel plot and Egger test conducted to detect publication bias, we do not think the present analysis was affected by publication bias. Owing to the small sample size of included studies and high heterogeneity across the included studies, the interpretation of rare events and safety analysis should be performed cautiously. A larger number of RCTs will have to be sampled in future meta-analyses to confirm our findings. Nevertheless, regardless of these limitations, our study applied rigorous methodology to compare SGAs and ETTs in patients undergoing laparoscopic surgery, and to our knowledge, this was the first systematic review of this topic.

In conclusion, we could not find any difference between SGAs and ETTs with respect to the rate of insertion success on the first attempt, and insertion time and OLP in patients undergoing laparoscopic surgery. However, the incidences of laryngospasm, cough at removal, dysphagia or dysphonia, sore throat, and hoarseness were higher in the ETT group than in the SGA group. Other complications showed no evidence of any difference between the SGA and ETT groups. Therefore, we presume that SGAs might be clinically useful as effective airways in laparoscopic surgery.

## Supplementary Material

Supplemental Digital Content
