# Supplementary material for: Comparison between supraglottic airway devices and endotracheal tubes in patients undergoing laparoscopic surgery: A systematic review and meta-analysis
Source: Medicine (Baltimore). 2016 Aug 19;95(33):e4598. doi: 10.1097/MD.0000000000004598 (PMC5370819; doi:10.1097/MD.0000000000004598)
Supplement: Supplemental Digital Content [file medi-95-e4598-s001.doc]

**Appendix**

**MEDLINE**

randomized controlled trial.pt

randomized controlled trial$.mp

controlled clinical trial.pt

controlled clinical trial$.mp

random allocation.mp

exp double-blind method/

double-blind.mp

exp single-blind method/

single-blind.mp

or/1–9

clinical trial.pt

clinical trial$.mp

exp clinical trial/

(clin$ adj25 trial$).mp

((singl$ or doubl$ or tripl$ or trebl$) adj25 (blind$ or mask$)).mp

random$.mp

exp research design/

research design.mp

or/11-18

10 or 19

Case report.tw.

Letter.pt.

Historical article.pt.

Review.pt.

or/21-24

20 not 25

exp Laparoscopy/

exp Hand-Assisted Laparoscopy/

laparoscop$.mp.

or/27-20

26 and 30

supraglottic airway.mp.

supra-glottic airway.mp.

SGA.mp.

SAD.mp.

extraglottic airway.mp.

extra-glottic airway.mp.

EGA.mp.

EAD.mp.

exp laryngeal masks/

laryngeal mask airway.mp.

LMA.mp.

(mask adj6 airway).mp.

(i-gel or i gel or igel).mp.

(air-Q or air q or airq).mp.

Aura.mp.

(cobra PLA or cobra perilaryngeal airway).mp.

Laryngeal tube.mp.

LT,mp.

Combitube.mp.

SLIPA.mp.

Esophageal blocker.mp.

Ambu auraonce.mp.

or/32-53

31 and 54

**EMBASE**

randomi?ed controlled trial$.mp.

'controlled clinical trial (topic)'/exp

controlled AND clinical AND trials

controlled clinical trial$.mp.

'randomization'/exp

'random allocation'/exp

random allocation.mp.

double-blind.mp.

single-blind.mp.

#1 OR #2 OR #3 OR #4 OR #5 OR #6 OR #7 OR #8 OR #9

'clinical trial (topic)'/exp

clinical AND trial$.mp.

random$.mp.

rct

#11 OR #12 OR #13 OR #14

#10 OR #15

'case study'/exp

'case report'/exp

'abstract report'/exp

'letter'/exp

#17 OR #18 OR #19 OR #20

#16 NOT #21

'laparoscopic surgery'/exp

Laparoscop$

laparoscope

laparoscopic

#23 OR #24 OR #25 OR #26

#22 AND #27

'supraglottic airway device'/exp

supraglottic airway.mp.

supra-glottic airway.mp.

SGA.mp.

SAD.mp.

extraglottic airway.mp.

extra-glottic airway.mp.

EGA.mp.

EAD.mp.

'laryngeal mask'/exp

laryngeal mask airway.mp.

'lma'/exp

i-gel or i gel or igel.mp.

air-Q or air q or airq.mp.

Aura.mp.

Cobra PLA

cobra perilaryngeal airway

Laryngeal tube

LT

Combitube

SLIPA.mp.

Esophageal blocker.mp.

Ambu auraonce.mp.

#29 OR #30 OR #31 OR #32 OR #33 OR #34 OR #35 OR #36 OR #37 OR #38 OR #39 OR #40 OR #41 OR #42 OR #43 OR #44 OR #45 OR #46 OR #47 OR #48 OR #49 OR #50 OR #51

#28 AND #52
